# Cecil: A Moment or a Movement? Analysis of Media Coverage of the Death of a Lion, *Panthera leo*

**DOI:** 10.3390/ani6050026

**Published:** 2016-04-25

**Authors:** David W. Macdonald, Kim S. Jacobsen, Dawn Burnham, Paul J. Johnson, Andrew J. Loveridge

**Affiliations:** Wildlife Conservation Research Unit, Department of Zoology, University of Oxford, Recanati-Kaplan Centre, Tubney House, Abingdon Road, Tubney, Oxfordshire OX13 5QL, UK; kimsjacobsen@gmail.com (K.S.J.); dawn.burnham@zoo.ox.ac.uk (D.B.); paul.johnson@zoo.ox.ac.uk (P.J.J.); tawnycat1@hotmail.com (A.J.L.)

**Keywords:** Cecil the lion, trophy hunting, media analysis, lions, viral, social media, editorial media

## Abstract

**Simple Summary:**

We provide chronology of events following the death of a lion nicknamed “Cecil” and analyse the global media coverage of the event spatially and temporally. We recruited a media monitoring company to explore patterns in both social and editorial media globally, regionally and by country. The number of articles in the editorial media mentioning Cecil the lion peaked at 11,788 on 29 July. There was remarkable global synchrony in this “spike”, so the world media appeared to respond as a globalised entity. We used media saturation, a relative measure of the number of mentions of the Cecil story, as a proxy for estimating the level of interest in the Cecil story. Regionally, saturation levels were high in North America. Interest was also high in Australia and parts of South America and Africa. This opposes the common assumption that interest in the Cecil story was the prerogative of wealthy nations. The social media response to Cecil’s death, was much larger than that in the editorial media in terms of the number of mentions of Cecil (87,533 mentions), but the time to the peak was very similar to that of the editorial media. We compared the development of coverage of the event in the three largest social media platforms (Facebook, Twitter and YouTube) to see whether they played identifiably different roles in the development of the story through time. All peaked at the same time, so there was no evidence that any one platform was responsible for precipitating the spread of the story in advance of the others. The editorial and social media also peaked in synchrony, neither one being a forerunner or follower in the coverage of the Cecil story. Instead, our results reveal a highly interconnected media universe: with the story going viral synchronously across media channels and geographically across the globe over the span of about two days. We consider whether the preoccupying interest in Cecil displayed by the millions of people who followed the story may betray a personal, and thus potentially political, value not just for Cecil, and not just for lions, but for wildlife, conservation and the environment. If so, then for those concerned with how wildlife is to live alongside the human enterprise, this is a moment not to be squandered and one which might have the potential to herald a significant shift in society’s interaction with nature.

**Abstract:**

The killing of a satellite-tagged male lion by a trophy hunter in Zimbabwe in July 2015 provoked an unprecedented media reaction. We analyse the global media response to the trophy hunting of the lion, nicknamed “Cecil”, a study animal in a long-term project run by Oxford University’s Wildlife Conservation Research Unit (WildCRU). We collaborated with a media-monitoring company to investigate the development of the media coverage spatially and temporally. Relevant articles were identified using a Boolean search for the terms Cecil AND lion in 127 languages. Stories about Cecil the Lion in the editorial media increased from approximately 15 per day to nearly 12,000 at its peak, and mentions of Cecil the Lion in social media reached 87,533 at its peak. We found that, while there were clear regional differences in the level of media saturation of the Cecil story, the patterns of the development of the coverage of this story were remarkably similar across the globe, and that there was no evidence of a lag between the social media and the editorial media. Further, all the main social media platforms appeared to react in synchrony. This story appears to have spread synchronously across media channels and geographically across the globe over the span of about two days. For lion conservation in particular, and perhaps for wildlife conservation more generally, we speculate that the atmosphere may have been changed significantly. We consider the possible reasons why this incident provoked a reaction unprecedented in the conservation sector.

## 1. Introduction

All actions have reactions, but some moments in history are defining. On 2 July 2015, the killing of a 13-year old male lion, *Panthera Leo* (nicknamed “Cecil” by the researchers in our team who had tracked his movements by satellite since 2009) certainly prompted a reaction, and we argue that, in terms of attracting global attention, it was the largest reaction in the history of wildlife conservation. As a moment, measured by media coverage and public engagement, it was immense. But was it a defining moment, in the sense of changing, or at least offering an opportunity to change, history? Will the Cecil Moment presage the Cecil Movement? 

As a step towards considering that larger question, here we document and quantify the unfolding story. As background, in 1999, two of us (David W. Macdonald and Andrew J. Loveridge), researchers at Oxford University’s Wildlife Conservation Research Unit (WildCRU) established a study of lions in Hwange National Park, Zimbabwe, and parts of the surrounding Kavango Zambezi (KAZA) landscape. The motivation was to provide evidence to underpin the conservation of the lions populating this region while advancing the well-being of the human communities living alongside them. Following an early focus on the impact of trophy hunting around Hwange on lions, our evidence resulted in a *ca*. 90% reduction of legally hunted lions as licensed by Zimbabwe’s National Parks and Wildlife Management Authority [1,2]. From 1999–2015, approximately 65 lions were hunted on the land surrounding the Protected Area, 45 of them were equipped with tracking devices. None of these deaths attracted much attention from the world’s media, including the two other satellite-collared lions, both also bearing nicknames, killed by trophy hunters in 2015. Similarly, the illegal shooting of a radio-collared cheetah nicknamed “Legolas” in October in Botswana scarcely gained a foothold in the press [3]. In considering why Cecil attracted such a volume of interest, we report the chronology of events. The salient facts are that Cecil, equipped with a satellite tracking collar, was reported (incorrectly) to have been lured by bait out of the park, wounded by the bowhunter (on an allegedly illegal hunt) who finally despatched the lion the next day. Alerted to this, the Zimbabwean National Parks and Wildlife Management Authority initiated an investigation of the trophy hunter, the professional hunter who accompanied him and the land-owner. Media coverage of this was picked up by U.S. talk show host, Jimmy Kimmel, who, on 28 July, broadcast an impassioned criticism of the episode. With the WildCRU web address on the screen, he said “show the world not all Americans are like this jackhole” (a reference to the dentist), telling his television audience about WildCRU’s work on this lion and urging them to support it by visiting our website. In the following h an estimated 4.4 million people did so, causing the collapse of both WildCRU’s and Oxford University’s websites. It is noteworthy, however, that media coverage of the story was showing a steep upward trend before the Kimmel monologue. In subsequent days the story spread explosively on both traditional and social media. For example, on 30 July there were some 900 articles worldwide about the WildCRU in 24 h.

Both the traditional editorial media, and, more recently, the social media, can contribute to social change and have been implicated in political upheavals [4] such as “the Arab Spring” [5]. Against this background, we seek to characterise the contagion of media reporting of the Cecil episode in both time, from day to day, and space, as it spread around the world. Our purpose is to document the episode both qualitatively and quantitatively. This is not only because it was widely felt to be unique in the history of conservation, but principally to begin the ambitious process of evaluating whether the global interest in the killing of this lion diagnoses a moment in history at which a wide citizenry revealed their disposition to value, and thus to conserve, wildlife more broadly. If so, this is important in the politics of conservation and the wider environment, and raises the possibility that the Cecil Moment might become the Cecil Movement.

## 2. Materials and Methods

### 2.1. Media Monitoring

Those of us who experienced the media onslaught, the print, visual and voice media outputs, the social media and the deluge of emails, phone calls and hits to our website, together with the interest of NGOs and government, have firsthand-qualitative experience: at the time David W. Macdonald and Dawn Burnham experienced this in Oxford, Andrew J. Loveridge in Zimbabwe. These impressions will form the basis of a discussion of the possible psychological mechanisms behind the global response and implications for the future of conservation. To quantify the contagion and character of media reports we identified a media-monitoring company that has one of the largest data bases of news and social media, Meltwater [6]. We selected them because of their reputation as a “highly regarded company serving academic research, government and “third sector” organisations” [7]. They can access 275,314 news sources globally and all public social media activity (Facebook, Twitter, YouTube, blogs, reviews, forums and more). 

### 2.2. Boolean Search

We identified relevant articles using a Boolean search for the terms Cecil AND lion. This search was repeated in 125 languages. These languages were identified from a pool of languages that combine the 100 most widely spoken [8] and the most influential [9] languages, yielding a combined total of 146. Twenty one of these languages, all of which might be regarded as obscure, were excluded because we could not adequately translate them, or because the Meltwater software could not process their script, leaving a sample of 125 surveyed languages (Supplementary materials, Table S1). 

Using these languages we interrogated the data base to reveal how mentions of “Cecil AND lion” occurred between the 1 July and 30 September, for both traditional editorial news media and social media. Each news article or social media item that mentions “Cecil” and “lion” (in any of the 125 languages) one or more times is registered as one “hit”. Hits are registered in Greenwich Mean Time (GMT). 

### 2.3. Estimating Media Saturation and Interest Levels

We use the number of hits divided by the number of publications screened in that country as a metric of media saturation. For the social media, we derive a measure of interest for each country by dividing the number of mentions of “Cecil AND lion” by the total number of internet users (internet usage data obtained from Graham *et al.,* [10]), assuming that the number of social media items mentioning a story occurs in proportion to the interest in that case among social media users.

### 2.4. Limitations

We are mindful that this approach is limited by the functionality of the Meltwater service. For example, we sampled only those editorial media sources that Meltwater searches (which Meltwater claims to be the most comprehensive selection in its sector). We also acknowledge the exclusion of some languages and the imprecision of the Boolean search term (although for editorial media the increase in instances of the search term jumping from an average of only 14.7 hits per day before Cecil was killed to up to 11,000 thereafter, gives us confidence that we are monitoring relevant stories). Further limitations include that the search can bring up only social media hits that are not protected by privacy settings and on YouTube can detect only words in the title, text and comment section, but not in the actual video clip itself. The number of social media hits originating within countries where Facebook, YouTube or Twitter are not used due to government restrictions on access, such as in China, is likely to be underestimated.

## 3. Results

### 3.1. Chronology of Developments in the Cecil Case

**Figure 1:**
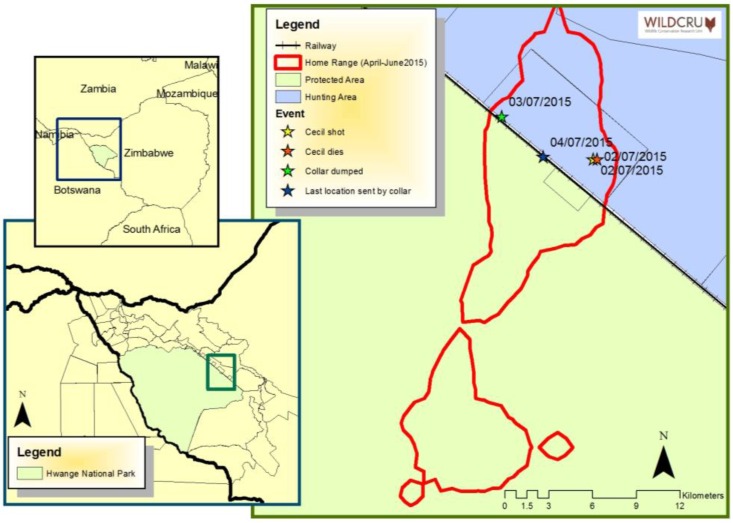
Map of the location of Hwange National Park within Zimbabwe (southern Africa map, small inset), map of the location of MAGM1’s (Cecil) home range within Hwange National Park (large inset) and detailed map of MAGM1’s home range (90% fixed Kernel, red outline) for three months prior to being hunted along with locations and dates of where the lion was first shot and wounded, where he was finally killed (approximately 250 m from where he was initially wounded), where the collar was dumped and the location from which the GPS unit transmitted its final position, prior to disappearing (presumed destroyed).

On 1 July 2015, at about 22:00, an American bowhunter, Walter Palmer, wounded the lion on Antoinette Farm, in the Gwaai Conservancy, directly adjacent to Hwange National Park. No hunting quota for 2015 had been issued for this area by Zimbabwe’s National Parks and Wildlife Management Authority to the owner, Honest Ndlovu, nor the professional hunter, Theo Bronkhorst, and so whether or not their client was complicit, the hunt was allegedly illegal. The hunters tracked the wounded lion and, allegedly, killed it with a second arrow the next day (about 11 h later). The lion was hunted on a bait, but was not lured from the Park as some media accounts have implied; the area was a part of the lion’s normal range. The lion was equipped with a satellite tracking device (Africa Wildlife Tracking, GPS Satellite collar) from which locational data were recorded every 2 h. Subsequently we have pieced together the most likely interpretation that the hunters moved the collar on the 3rd from the point where Cecil was killed, discarded it before returning on the 4th to move it again. At which point the collar stopped working, we presume because it was destroyed (as frequently happens when a study animal is killed illegally). This interpretation of events was corroborated by the subsequent investigation by Zimbabwe National Parks and Wildlife management and is consistent with the satellite data that we have downloaded (Figure 1).

**Figure 2:**
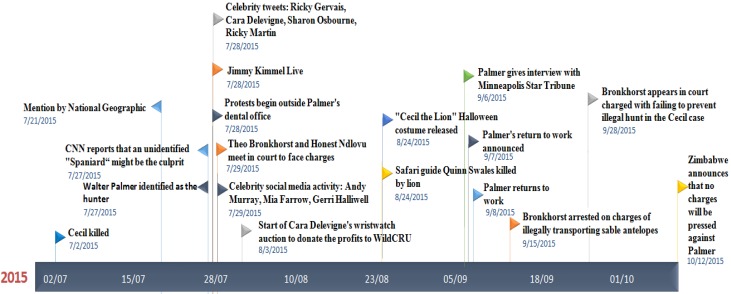
Time-line of events following Cecil’s death.

**Figure 3:**
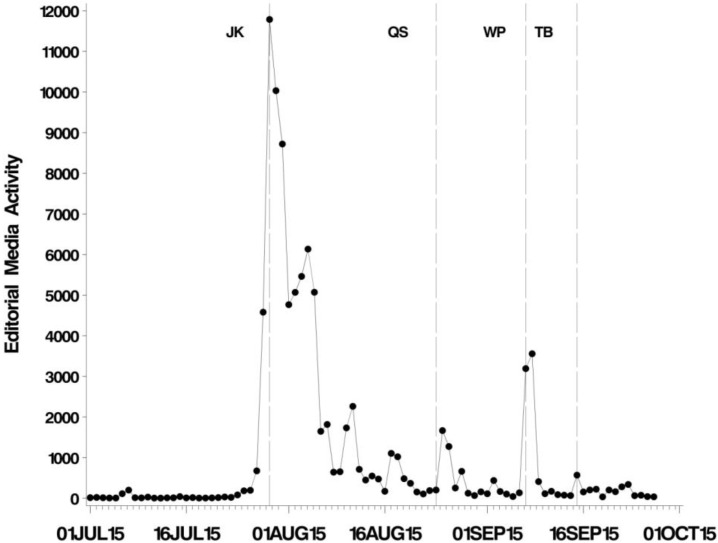
Temporal pattern in editorial media activity. Annotated lines indicate events coinciding with discernible spikes in coverage. JK: Jimmy Kimmel’s broadcast. QS: Death of Quinn Swales. WP: Announcement of Walter Palmer’s return to work. TB: Theo Bronkhorst arrested on charges of smuggling sable antelopes.

**Figure 4:**
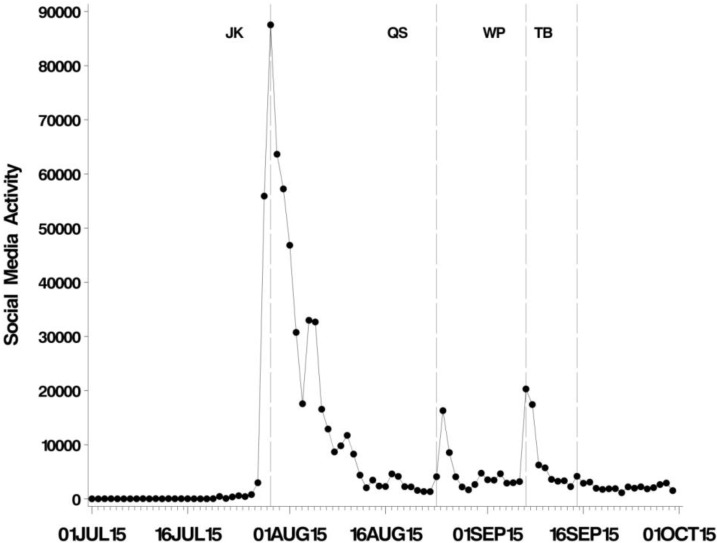
Temporal pattern in social media activity. Annotated lines indicate events coinciding with discernible spikes in coverage. JK: Jimmy Kimmel’s broadcast. QS: Death of Quinn Swales. WP: Announcement of Walter Palmer’s return to work. TB: Theo Bronkhorst arrested on charges of smuggling sable antelopes.

Key dates in the unfolding story are given in Figure 2. On 27 July, Walter Palmer was named as the hunter who killed Cecil, and on 28 July, protests erupted outside his clinic in Minnesota. On 28 July, Jimmy Kimmel held his monologue on ABC (The American Broadcasting Company). Quinn Swales, a safari guide working in Hwange national park was killed by another lion in a different part of Hwange National Park on 24 August, creating a spike in the media coverage mentioning Cecil (Figure 3 and Figure 4). The “Lion Killer Dentist Halloween Costume”, a controversial Halloween costume inspired by the killing of Cecil, was released on 25 August. On 7 September, the announcement that Walter Palmer would return to work was made, and he returned to work on 8 September and we observe a distinct spike in media coverage (Figure 3 and Figure 4). On 15 September, Theo Bronkhorst was implicated in the smuggling of sable antelopes.

### 3.2. The Development of Coverage in the Editorial Media

Following a period of background hits (some of which are attributable to Cecil’s fame in the photo-tourist arena, and some perhaps to spurious matches to the Boolean search), for the three weeks following the lion’s death the hit rate was increasing. But on 29 July, media coverage exploded to a peak of 11,788 hits (Figure 3). Between 1 July and 30 September there were a total of 94,631 hits in the editorial media.

The total level of media saturation for the period we surveyed varied among regions, *F*_5, 109_ = 25.4, *p* < 0.001), being highest in North America, and lowest in the Middle East (Supplementary materials, Figures S1–S7). The regional responses in the editorial media show a high level of similarity in the timing of the peak media saturation (Supplementary materials, Figures S1–S7). The peaks of coverage, occurred at around the same time: the mean lag in days from the start of the sampling period (1 July) to peak coverage was 29.2 days (CI 28.8–29.5) and there was no evidence that peak coverage varied among regions (*F*_6, 146_ = 1.5, *p* = 0.18, all analyses weighted by total number of hits for each country). In short, in terms of timing, as distinct from magnitude of interest, the world responded as a globalised entity. Some of the subsequent smaller ‘spikes’ of media saturation show less consistent timing, being clear in some regions but not others. For example there are two clear peaks at the end of August and around mid-September that are clear in Europe and North America, but much less so in Oceania, Asia and Africa.

There was significant variation among the world regions in the level of sustained media saturation after the peak (*F*_6, 146_ = 13.6, *p* < .0001). This was defined as the proportion of days after the day of peak coverage when coverage remained at least at 10% of the peak (Supplementary materials, Figures S1–S7). This was highest in Africa and lowest in Oceania and Europe. We observed that the number of internet users in a country increased in proportion with population size (slope on log-log scale = 1.01, SE = 0.04), but declined with infant mortality strongly suggesting lower access in less developed countries (slope on log-log scale = −0.71, SE = 0.05). Similarly, the country level number of publications searched by Meltwater also increased with population size, though less steeply than in proportion with population size (slope on log-log scale = 0.79, SE = 0.05), and declined more steeply than in proportion with infant mortality (slope on log-log scale = −1.34, SE = 0.07), suggesting a greater effect of development on availability of editorial media.

### 3.3. The Development of Coverage in the Social Media

The social media record much higher volumes of hits (peak: 87,533 hits on 29 July) compared to editorial media, with a total of 695,983 hits between 1 July and 30 September (Figure 4). However, the trajectories of social and editorial media coverage were very similar.

**Figure 5:**
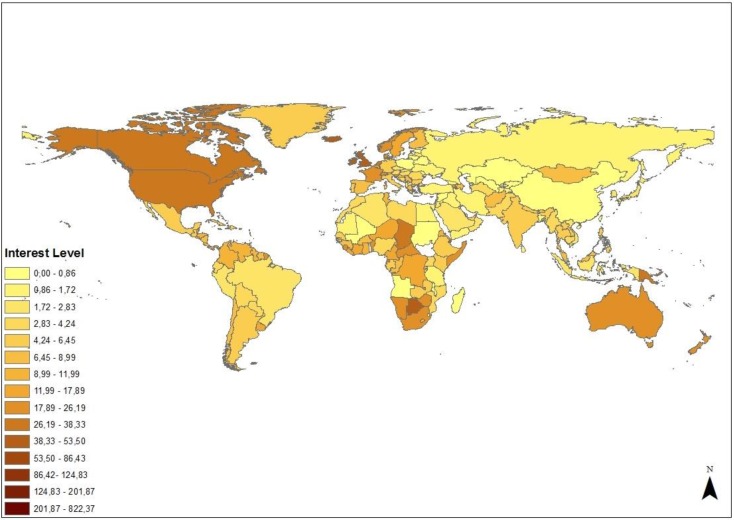
Levels of interest revealed by social media. Relative interest values were multiplied by 100,000 to facilitate representation in ArcGIS.

In order to gain a measure of the level of interest in Cecil across the globe we calculated relative interest. High levels of relative interest were recorded in North America, Australia and Western Europe (Figure 5). Relative interest was also high in several countries in Southern Africa, not just Zimbabwe.

### 3.4. The Response to Cecil the Lion’s Death on Different Social Media Platforms

Between 1 July and 30 September, Twitter recorded 70,219 hits, Facebook 9961 and YouTube 185. Peaks for all platforms occur on 29 July (Supplementary materials, Figures S8–S10). The chronologies are very similar for Twitter and Facebook, whereas that for YouTube remains high for two days after the peak and thereafter remains proportionally higher.

## 4. Discussion

The attention given to the Cecil story was immense, reaching 99,000 reports per day in the social and editorial media combined (nearly 1000 per day mentioning WildCRU). Strong feelings were clear in the content of social media postings. And in the non-digital world, protests took place outside Mr. Palmer’s dental surgery. Those protests started before Jimmy Kimmel delivered his monologue. The patterns are closely similar between the editorial and social media, and in both cases the huge peak of interest declines over the course of about one month. While thereafter the daily scores seem small in comparison to the peak, they remain large by comparison with most conservation stories. At the least, the Cecil Moment is a prolonged one. 

The editorial media peaked in synchrony across the world’s regions but after the peak there were differences. Media saturation was highest in North America, the home of the trophy hunter dentist, and where the Jimmy Kimmel appealed to patriotism. The Cecil story showed the most dramatic behaviour in this region. In contrast, it took longer before Africa experienced its largest increase in hits from one day to the next, and had a more sustained interest level after the peak, with fewer spikes defined as events. Thus, the coverage of the Cecil story seems to have been more moderate and more sustained in Africa compared to other world regions.

The coverage in both editorial and social media was similar. The peaks occurred at the same time, and there were no obvious differences in the trajectories. This does not support assumptions that viral phenomena of this nature are primarily driven by social media (we use the expression “viral” in the same sense as Berger and Milkman 2012) [11]. Also when comparing three of the largest social media channels to each other the synchrony is striking. There are no clear forerunners within the social media sphere or between editorial and social media, suggesting instead a highly interconnected media universe: this is the reality with which conservationists must interact, and involved Cecil’s story spreading synchronously across media channels, and geographically across the globe, over the span of about two days.

The distribution of social media interest across the globe (Figure 5) indicates that interest in the Cecil case was not limited to wealthier countries. There is a high level of engagement across certain sub-Saharan countries and the interest levels in large parts of South and Central America are comparable to those in Europe. This conclusion is further supported by the observation that media saturation in each country was unrelated to infant mortality (data for 2011–2015 obtained from The World Bank [12]) which is an indicator of population health [13], or to the indices of governance, economic success or conservation policy derived by Dickman *et al.* [14] (|r| < 0.15, *p* > 0.07). This has to be interpreted in light of the fact that a significant portion of the population in many of these countries will have limited access to internet or printed media, so the media saturation/interest levels will be determined by the segments of society that do.

We cannot say exactly which aspect of the case was attracting attention at a given time in a given place or, for example, whether it was motivated by distaste for trophy hunting or concern for either animal welfare or conservation (we are mindful that further content analysis, which we plan, could reveal much about the motivations of underlying the public and media responses). Hits may also be generated by people expressing opposing views or denouncing the media attention given to Cecil’s death (e.g., [15]), so we can only document the degree to which the editorial media and social media users in a country engage in coverage and debate about Cecil’s death.

Why did this particular story touch a wide public so forcefully when such trophy hunting events are far from unusual? The lion in question was majestic, rather well-studied, bore an English nickname (more memorable than its data code of MAGM1); it was allegedly lured to its death (although it routinely left the park of its own volition), was wounded with an arrow and endured a lingering death, was killed in apparently dubious circumstances, by a client who was identifiable (as opposed to amorphous villains such as polluting industries in the case of climate change), wealthy, white, male and American and who, to judge by media reports, had previously been associated with problematic hunting episodes. Each of these elements might seem problematic and, to some, not just reprehensibly illegal but morally deplorable [16], but neither separately nor in many partial combinations are they unique. It seems plausible then that it is their combination, certainly unusually and perhaps uniquely, that led to the viral explosion. It might also be the case that the episode was so remote from the daily experience of many people, that they felt able to make a straightforward condemnation of it, with no feeling of related guilt about their own behavior [17]. Studies of the forces that shape viral episodes online have found that physiological arousal is important [11]. Negative emotions increase transmission rates – but anger is a more potent stimulant for sharing posts than is sadness because it is accompanied by greater arousal. It is clear from the most cursory exploration of the social media content that visible anger was a very common thread running through posts concerning Cecil. Something else that may have contributed to the virality of the episode was its breadth of appeal. Surprise and anger were not confined to any geographic region. There is some evidence that, while cascades of “sharing” on social media are difficult to predict, breadth in the sense of the number of ‘roots’ a re-sharing tree has is a good predictor of large cascades [18]. We might compare the Cecil episode to that which surrounded a man-eating tiger in India [19]. A decision to relocate the tiger from a National Park caused very different reactions across Indian society, with strong feelings and anger on both sides of the debate, and wide coverage on social media. However, the debate attracted little coverage outside India. There is some evidence that negative stories have lower persistence than positive ones [20]; the trajectory of the ‘Cecil’ story coverage fits more closely that of the ‘bad news’ than ‘good news’ stories studied by those authors. 

Although we have not quantified the content of the media coverage or the opinions expressed in the social media, we have gained impressions from these, and also from direct correspondence to our Unit, about why the hunt provoked the anger that it did. For example, from the many emails and letters, we inferred that people were more concerned that the hunt was for a trophy rather than for meat, that the quarry was a carnivore rather than an ungulate, and that it was a large species rather than a small one. There was overwhelming distaste for trophy hunting of a big cat, and a sense that this approbation was fuelled by moral indignation at the act, generally articulated more in terms of a concern for animal welfare (including the family factor of concern for the surviving pride members) than for conservation. These motivations, and particularly the perceived framing of distaste in terms of animal welfare, and possible conflation between this topic and conservation, are subjects that merit further research. It was clear from many interviewers and correspondents that there was widespread ignorance that trophy hunting still occurs widely across Africa, or that much more land is in fact set aside for trophy hunting than for National Parks, or that some mainstream conservationists believe that properly managed trophy hunting can contribute to conservation [21], even if it is uncertain how often it does so [22]. 

Indeed, the suggestion that trophy hunting of lions could be a tool for conservation (a proposition that requires urgent critical analysis), through giving commercial value to animals that in some parts of Africa would otherwise be considered as pests and not tolerated, was seemingly widely regarded as morally despicable. This raises interesting and urgent societal (and ethical) issues: not just in the wider context of wildlife conservation, but specifically in the case of lions and other big predators. The revenues of both trophy hunting and photo-tourism are widely discussed, and often said to be not only engines for conservation and incentives for maintaining wildlife habitat instead of conversion to alternative land-uses, but also a driver for the development of poor communities. In the case of lion economics, these claims merit more careful analysis than has hitherto been the case. Our team has identified, and is now tackling, two critical questions: to what extent would the cessation of trophy hunting for lions cause a reduction, or even collapse, of the financial support that trophy hunting makes to wildlife conservation, and thus what sum of money would be required to make good the loss of revenue to conservation should trophy hunting of lions cease? It is urgent to quantify this figure insofar as an alternative source for it would have to be found in the event that trophy hunting of lions ceased. Insofar as global opinion might create circumstances which made lion trophy hunting unviable (or banned it outright), and insofar as such hunting might indeed currently contribute to financing conservation of lions and beyond, it would seem prudent to plan for a journey towards that outcome, rather than to jump precipitously to it. That said, our sense of the global interest in Cecil was that it suggested a prevailing moral standpoint whereby ethical consideration rendered it inappropriate to consider economic ones. We sensed that the prevailing opinion was that ethics trumped finance in this context, and that arguing against that was perceived as morally equivalent to, for example, justifying slavery on the basis of its revenue generating capacity. Conservationists seeking the best consequentialist compromise, then, were likely to be judged harshly by history. If this is indeed the prevalent global mood, and insofar as trophy hunting does indeed give lions a value that prevents their eradication, it is obviously urgent to find an alternative means of encouraging (presumably paying) people to tolerate them In addition to media attention, which costs the individuals involved almost nothing, by 25 September 2015, WildCRU had received $1.06 million dollars from 13,335 donors (the majority from North America). We can assume that these were a persistent subset of the 4.4 million visitors to the WildCRU website on the night of the Jimmel Kimmel plea that were thwarted in their attempts to donate. If conservationists are able to harness this enthusiasm and action then perhaps there is hope that global society could pay for global commons and secure the protection of lions in Africa. Indeed, the particular charismatic appeal of big cats [23], combined with the unique response to Cecil, in the face of the species’ rapidly deteriorating status [24], suggest to us that lions might have a unique capacity to galvanize populist funding from the public to foster their own conservation and thereby to act as ambassadors for wildlife more widely.

The urgency to find means to make good any loss of funds for lion conservation that might follow from a reduction or cessation in trophy hunting is enhanced by the radical shifts in related policy that have already been prompted by Cecil’s death. For example the banning of transporting lion trophies from Africa to Europe and America by several airlines [25] may precipitate, very quickly, a state change in the viability of lion trophy hunting. Similarly, the requirement of the USFWS amendments to the Endangered Species Act, requiring nations to demonstrate that lion hunting is managed in ways that give a net advantage to lion conservation may set a bar that cannot easily be jumped, and it would not be surprising if similar strictures were adopted by the European Union [26]. In short, insofar as lion conservation is affected by trophy hunting, the conservation community would be wise to prepare for rapid change. This is a moment that offers an opportunity for radical thinking about the conservation of lions, and indeed of wildlife more generally: It raises a deep question that is ultimately about how, in the 21st Century, the human enterprise is to co-exist with nature.The answers will lie beyond conservation biology and, while fostering the very best that existing approaches can, and may yet offer, will require innovative thinkers to stand back, seek new strategic ideas and ask: can the mold be broken? The answers are likely to lie at the interface of cutting edge economics, development, governance, regulation and, ultimately, politics. In particular, does the global interest in lion conservation unleashed by the killing of this lion create a moment, the Cecil Moment, that can be grasped to enable conservationists to lead wider society in stopping, and indeed reversing, the decline in lions? Maybe there is no such opportunity, given the pressures of a burgeoning human population set to double in Africa by 2050 [27]; but, there is a chance that the opportunity does exist and, fearful of squandering it, now is the moment to take a fresh look at high level strategic thinking for lion conservation.

## 5. Conclusions

**Figure 6:**
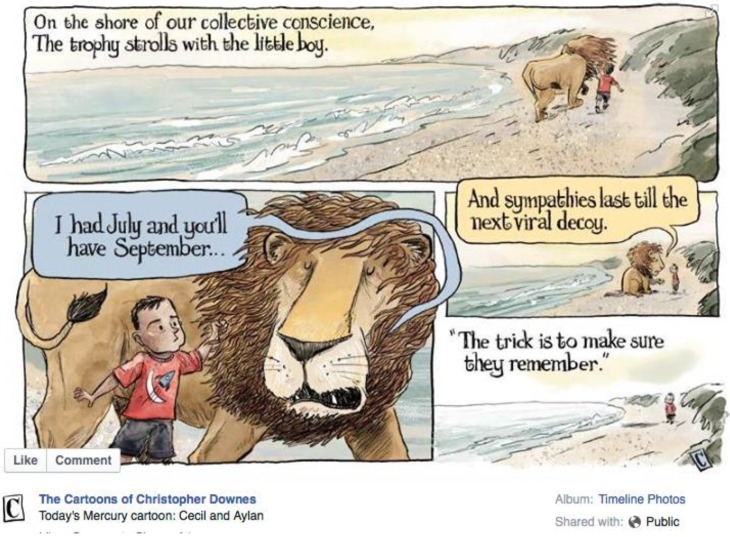
Mercury cartoon by Christopher Downes (8 September 2015).

So, whatever the explanation for the explosive spread of editorial and social media stories about the killing of this lion, and whatever the unusual durability of the story and its extraordinary penetration to all walks of life all over the world, the big question is whether this is merely an ephemeral phenomenon, and whether the public interest it reveals transcends the details of the episode. A poignant cartoon in the New Yorker prompted by the tragic drowning of a youthful Syrian refugee, Alan Kurdi, which briefly captured media attention a month after Cecil’s death, implied that all such stories wash up on the beach of societal consciousness, but are swept away at the next tide (Figure 6). Alternatively, it may be that the preoccupying interest in Cecil displayed by the millions of people who followed the story betrays a personal, and thus potentially political, value not just for Cecil, and not just for lions, but for wildlife, conservation and the environment. If so, then for those concerned with how wildlife is to live alongside the human enterprise, this is a moment not to be squandered and one which might have the potential to herald a significant shift in society’s interaction with nature. Could those millions of citizens transform the Cecil Moment into the Cecil Movement?

## Supplementary Materials

The following are available online at http://www.mdpi.com/2076-2615/6/5/26, Table S1: List of languages, Figure S1: Hits over time in Africa, Figure S2: Hits over time in Asia, Figure S3: Hits over time in Oceania, Figure S4: Hits over time in Central and South America, Figure S5: Hits over time in Europe, Figure S6: Hits over time in Middle East, Figure S7: Hits over time in North America, Figure S8: Hits over time on Twitter, Figure S9: Hits over time on Facebook, Figure S10: Hits over time on YouTube.

## Abbreviations

The following abbreviations are used in this manuscript:

ABC	The American Broadcasting Company
WildCRU	Wildlife Conservation Research Unit
KAZA	Kavango Zambezi
GMT	Greenwich Mean Time
USFWS	U.S. Fish and Wildlife Service
