# Supplementary material for: Cecil: A Moment or a Movement? Analysis of Media Coverage of the Death of a Lion, Panthera leo
Source: Animals (Basel). 2016 Apr 25;6(5):26. doi: 10.3390/ani6050026 (PMC4880843; doi:10.3390/ani6050026)
Supplement: Supplementary file 1 [file animals-06-00026-s001.pdf]

Supplementary Materials: Cecil: A Moment or a Movement? Analysis of Media Coverage of the Death of a Lion, *Panthera Leo*

David W. Macdonald, Kim S. Jacobsen, Dawn Burnham, Paul J. Johnson and Andrew J. Loveridge

1. List of Languages

Table S1. List of languages.

Languages Searched by Meltwater News	Languages Unable to be Searched by Meltwater News
Afrikaans	Amharic
Akan	Bhojpuri
Albanian	Burmese
Arabic	Chhattisgarhi
Armenian	Deccan
Assamese	Dhivehi
Asturian	Gujarati
Awadhi	Haryanvi
Azerbaijani	Kannada
Balochi	Khmer
Basque	Lao
Bavarian	Madurese
Belarusian	Malayalam
Bengali	Marwari
Breton	Mossi
Bulgarian	Oriya
Cantonese	Punjabi
Catalan	Sinhalese
Cebuano	Sylheti
Central Khmer	Telugu
Chewa	Tibetan
Chinese	
Chittagonian	
Colognian	
Czech	
Danish	
Dhundhari	
Dutch	
Emiliano-Romagnolo	
English	
Esperanto	
Estonian	
Faroese	
Fiji Hindi	
Filipino	
Finnish	
French	
Fula	
Galician	
Gan	
Georgian	
German	
Greek	
Haitian	
Hakka	
Hausa	
Hebrew	
Hiligaynon	
Hindi	
Hmong	

Table S1. *Cont.*

Languages Searched by Meltwater News	Languages Unable to be Searched by Meltwater News
Hungarian	
Icelandic	
Ido	
Igbo	
Ilokano	
Interlingua	
Irish	
Italian	
Japanese	
Javanese	
Kazakh	
Kinyarwanda	
Kirundi	
Konkani	
Korean	
Kurdish	
Latvian	
Limburgan	
Lithuanian	
Lombard	
Luxembourgish	
Macedonian	
Magahi	
Maithili	
Malagasy	
Malay	
Maltese	
Marathi	
Min bei	
Min dong	
Min nan	
Mongolian	
Neapolitan	
Nepali	
Norwegian	
Occitan	
Oromo	
Pashto	
Persian	
Polish	
Portuguese	
Quechua	
Romanian	
Romansh	
Russian	
Saraiki	
Scottish Gaelic	
Serbo-Croatian	
Shona	
Sicilian	
Sindhi	
Slovak	
Slovenian	
Somali	
Spanish	
Sundanese	
Swahili	
Swedish	
Tamil	
Thai	
Turkish	
Turkmen	

Table S1. *Cont.*

Languages Searched by Meltwater News	Languages Unable to be Searched by Meltwater News
Ukrainian	
Urdu	
Uyghur	
Uzbek	
Vietnamese	
Welsh	
Wu	
Xhosa	
Xiang	
Yiddish	
Yoruba	
Zhuang	
Zulu	

2. Hits over Time in the Editorial Media

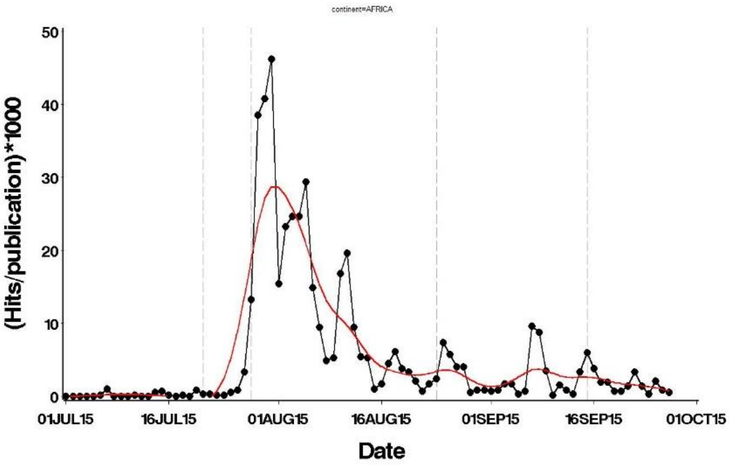

Figure S1. Hits over time in Africa.

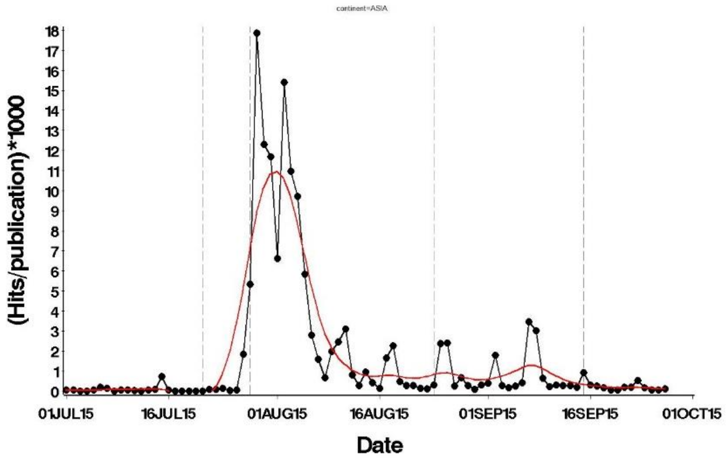

Figure S2. Hits over time in Asia.

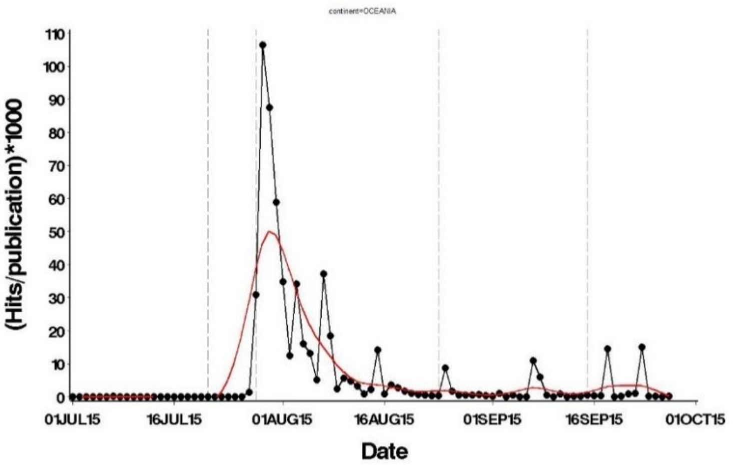

Figure S3. Hits over time in Oceania.

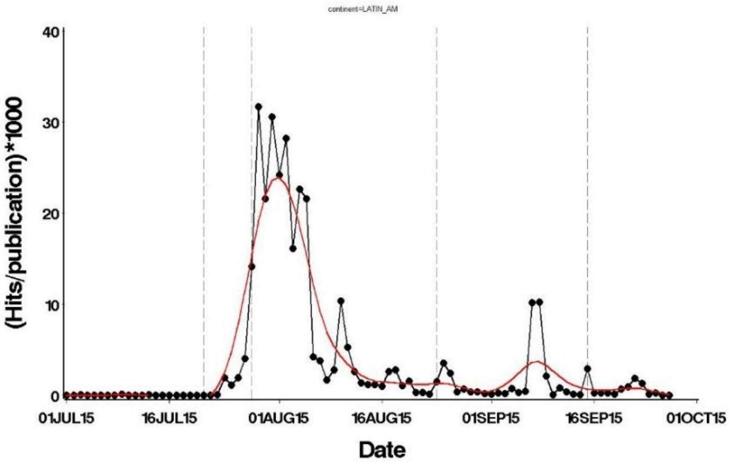

Figure S4. Hits over time in Central and South America.

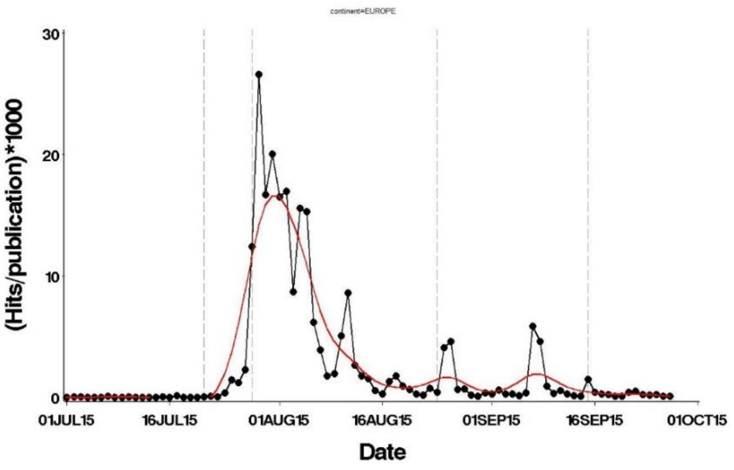

Figure S5. Hits over time in Europe.

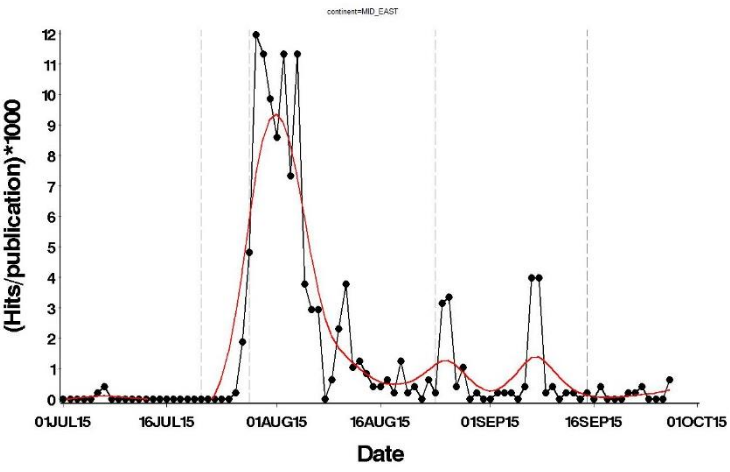

Figure S6. Hits over time in Middle East.

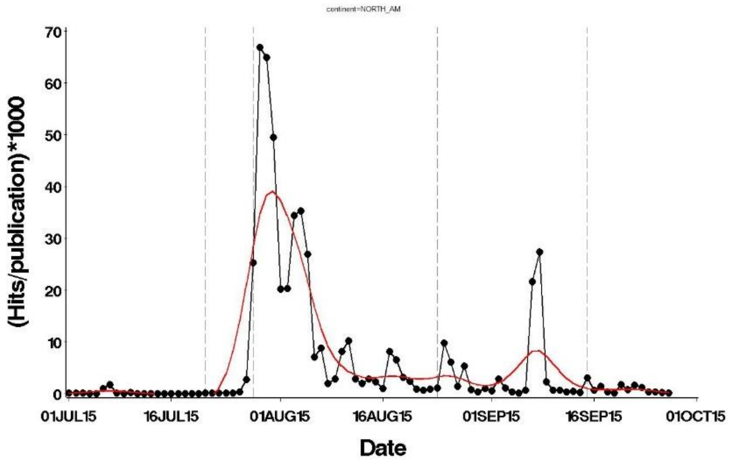

Figure S7. Hits over time in North America.

3. Hits over Time on Twitter, Facebook and YouTube

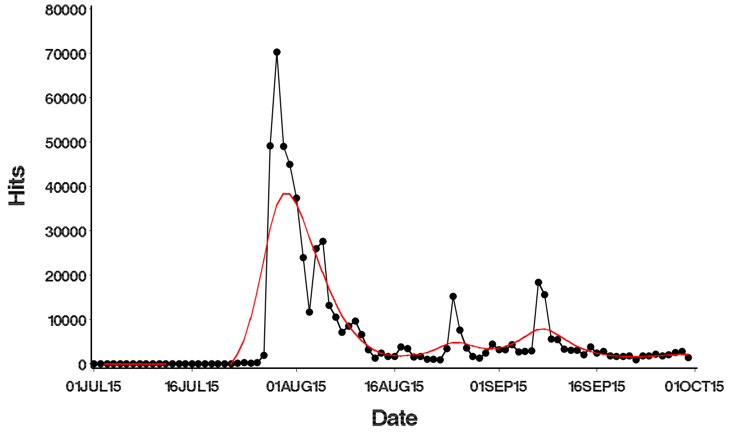

Figure S8. Hits over time on Twitter.

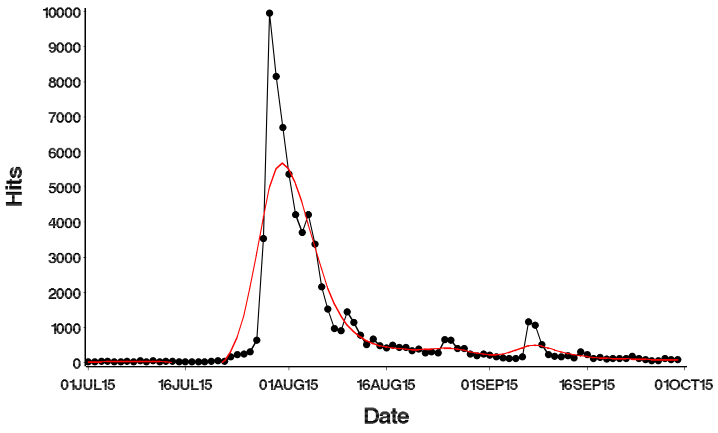

Figure S9. Hits over time on Facebook.

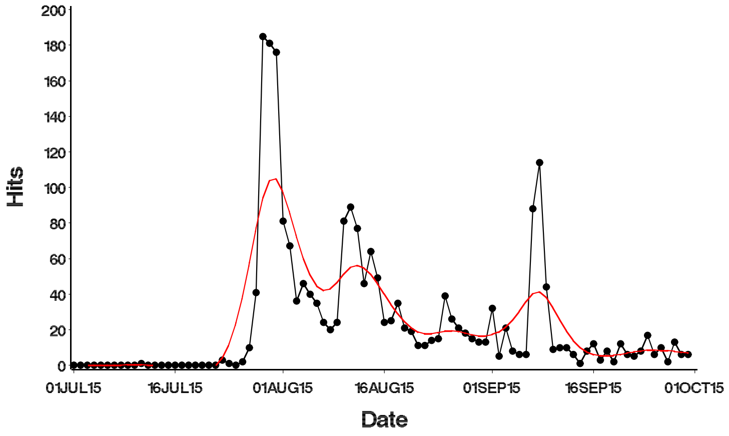

Figure S10. Hits over time on YouTube.

© 2016 by the authors; licensee MDPI, Basel, Switzerland. This article is an open access article distributed under the terms and conditions of the Creative Commons by Attribution (CC-BY) license (http://creativecommons.org/licenses/by/4.0/).
